# Social Implications of Malaria and Their Relationships with Poverty

**DOI:** 10.4084/MJHID.2012.048

**Published:** 2012-08-09

**Authors:** Francesco Ricci

**Affiliations:** Infectious Diseases Department, G. d’Annunzio University of Chieti.

## Abstract

In poor countries, tragically, people die unnecessarily. Having changed our understanding about issues related to poverty, even in the fight against malaria we must keep in mind a number of issues other than simple lack of economic resources. In this article we tried to discuss the various aspects that make malaria a disease closely related to poverty and the effects of malaria on the same poverty of patients who are affected. If you want the program to "Rool Back Malaria" to succeed, you must program interventions that improve the living conditions of populations in endemic area, individually and as communities. As has become clear that the discovery of an effective vaccine will not eradicate the disease, remains a fundamental understanding of mechanisms related to poverty that cause Malaria remains one of the major killers in the world, to help communities affected and individuals to prevent, cure properly and not being afraid of this ancient disease.

## Introduction

In poor countries, tragically, people die unnecessarily.^1^ This is a concept known and recognized throughout the world that the inhabitants of more developed and rich countries have a better life expectancy compared to the poorest countries. The reasons are not only linked to health care costs that often reflects health systems most technologically advanced and rich resources.^2^

Over the past two to three decades, our understanding of poverty has broadened from a narrow focus on income and consumptions to a multidimensional notion of education, health. social and political participation and rights, personal security and freedom, and environmental quality.^2^,^3^ Thus poverty encompasses not just low income, but lack of access services, resources and skills, vulnerability, insecurity, voicelessness and powerlessness. Multidimensional poverty is a determinant of health risks, health-seeking behavior, health care access and health outcomes.^3^,^4^

An estimated 70% of the world’s poor are women.^3^ Similarly, in the Western Pacific Region, poverty often wears a woman’s face. Indicators of human poverty, including health indicators, often reflect severe gender – based disparity. In this way, gender inequality is a significant determinant of health outcomes in the Region, with women and girls often at a severe social disadvantage.^2^

Malaria remains a global public health problem. Approximately 40% of the world’s population lives in more than 140 countries at risk of malaria. In the Western Pacific Region, Malaria is endemic in 10 countries. In Africa malaria is endemic in more than 30 countries (see figure 2)^5^.^4^,^5^

The strategies employed to prevent and control malaria have been effective in reducing the burden of disease in many countries. Yet, as analyses of health outcomes become more refined, it is increasingly evident that poor and marginalized populations might not be benefiting from investments in malaria prevention and control.

In areas of high malaria transmission (stable transmission areas), repeated malaria infections provide inhabitants with partial immunity. In contrast, unstable malaria areas are characterized by outbreaks and irregular epidemics among people with low immunity. In stable and unstable areas, pregnant women and children under 5 years old are at greatest risk of the most severe clinical symptoms of malaria. This is because a woman’s immunity is temporarily depressed during pregnancy, while the immune system of small children is not fully developed.^3^

## Overview of Burden of Disease

**Figure 1:**
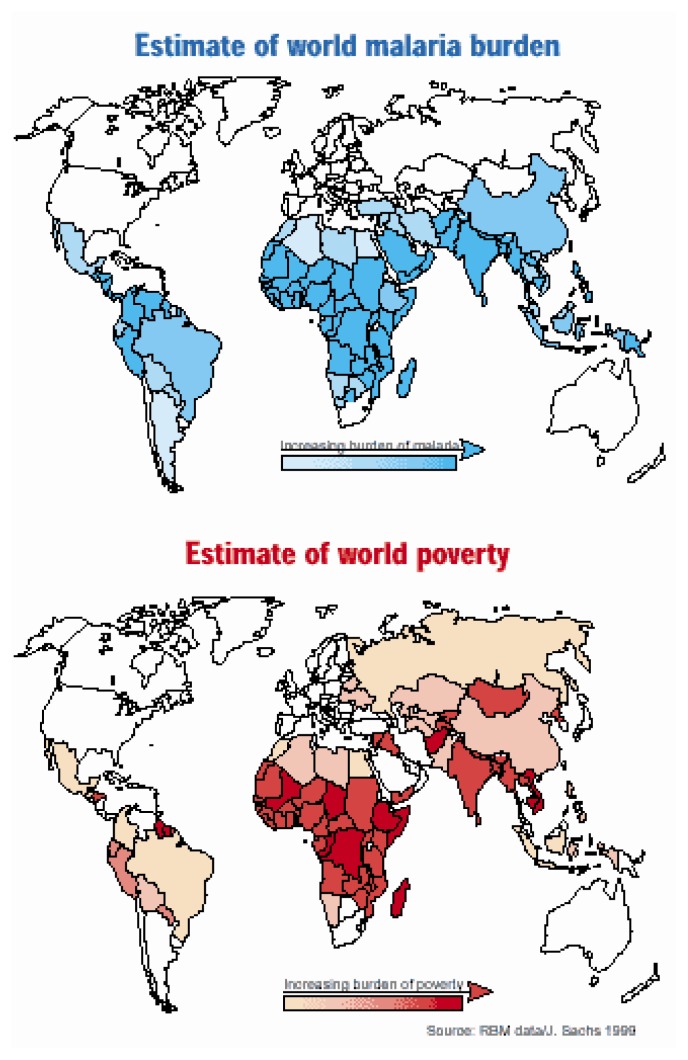
Relationship between the burden of malaria worldwide and poverty levels estimated in the world

**Figure 2:**
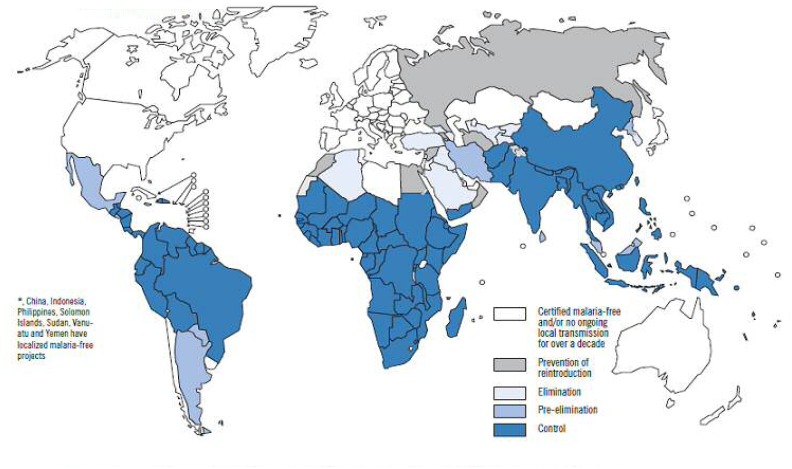
Malaria-free countries and malaria-endemic countries in phases of control*, pre-elimination, elimination and prevention of reintroduction, end 2008

Epidemiological data of the disease are discussed in an other session of this paper. Table 1 and Figure 2 show data from the WHO 2009 Malaria Report.^5^ As you can see, while malaria is not exclusively a disease of the poor, the deprivation associated with poverty can increase the risk of malaria. Figure 1^6^ shows the relationship between estimate of word malaria burden and estimate of world poverty. The relationship between malaria and poverty plays out along a number of distinct, yet interrelated, pathways. Poorer and marginalized communities might be more likely to suffer from malaria than non less poor communities, because their geography and environment are more hospitable to mosquitoes than areas inhabited by non-poor communities.^3^

Poverty also might reduce the likelihood that households will adopt appropriate preventive measures (such as sleeping under an insecticide treated net [ITN]) and curative measures (seeking timely health care for fevers). This can result in greater malarial morbidity and mortality among the poorer than the non-poor. Conversely, malaria might further impoverish poorer households through the costs of preventive and curative measures, as well as for the inability to work while ill.

Importantly, because gender and poverty interact to produce unique disadvantages among poorer women, gender is considered an independent risk factor.

## Defining Poverty

Poverty is increasingly considered multidimensional and its definition goes well beyond the narrow association of poverty with low income and consumption. Poverty encompasses other forms of deprivation, including economic opportunities, education and health outcomes, access to services, and resources and skills. This definition also covers additional aspects, such as voicelessness, vulnerability and powerlessness to influence decisions that affect their lives. In the Pacific areas, for example, income or consumption poverty tends to be low or nonexistent, but households there can be vulnerable to natural disasters; be isolated or remote; lack economic choices (or opportunities to earn a cash income); have limited access to educational, health and financial services; and suffer from social exclusion.^7^

Members of the same household tend to experience poverty differently, depending on factors such as gender, age and marital status. Women tend to be particularly disadvantaged. The United Nations Development Programme (UNDP) estimates that 70% of the world’s poor are women.^7^ In addition, women lag behind men in almost every social and economic indicator of well-being.^8^

## Malaria and Poverty

### Inequalities in Incidence

An estimated 58% of malaria deaths occur among the poorest 20% of the world’s population.^9^ The inequality of this distribution is higher than that for any other disease of public health importance. In a very recent study from Ghana,^10^ 1496 children presenting to the hospital were examined for malaria parasites and interviewed with a standardized questionnaire. The information of eleven indicators of the family’s housing situation was reduced by a Principal Component Analysis (PCA)^11^ to a socioeconomic score, which was then classified into three socioeconomic statuses: poor, average and rich. Their influence on the malaria occurrence was analyzed together with malaria risk co-factors, such as sex, parent’s educational and ethnic background, number of children living in a household, applied malaria protection measures, place of residence and age of the child and the mother. The multivariate analysis demonstrated that the proportion of children with malaria decreased with increasing socioeconomic status as classified by PCA (p < 0.05). Other independent factors for malaria risk were the use of malaria protection measures (p < 0.05), the place of residence (p < 0.05), and the age of the child (p < 0.05). The socioeconomic situation is significantly associated with malaria even in endemic rural areas where economic differences are not much pronounced.

### Low Household Income

Low income and consumption are important aspects of poverty.^3^ Poor households and individuals are prevented from consuming goods and services that otherwise would protect them against the risks of malaria. A literature review was undertaken in 2003 to critically assess evidence on malaria incidence or vulnerability to the effects of malaria.^12^ Citing studies from countries worldwide, the review concluded that the poorest countries suffer the greatest burden of malaria. However, evidence from household - and community - level case studies that stratified data along socioeconomic lines, present conflicting pictures of the distribution of malaria incidence among poor and less poor households.

In sub-Saharan Africa, a link between low income and the incidence of fever has been observed at the district level.^13^

### Social Exclusion

An important aspect of poverty is that it often overlaps with, and reinforces, other types of social exclusion (such as those based on race, ethnicity, geographic location urban/rural and gender) that perpetuate inequalities. The social exclusion of ethnic groups is often reflected in the relatively lower levels of development and higher rates of poverty in the areas where they live.^3^

### Housing

For the poor, living conditions are often characterized by inadequate housing and overcrowding, which can increase the risk of malaria. Dwellings that are hastily constructed, or made of readily available materials, might allow mosquitoes to enter more easily than well-constructed housing with screened windows, thus increasing vector contact.^14^ Some evidence suggests that overcrowding might increase the risk of malaria, because mosquitoes are attracted by the higher concentration of carbon dioxide and other chemicals in crowded houses.^7^

Family living space also might not be adequately separated from domestic animals, and the animals’ body temperature might attract mosquitoes.^14^

In a recent survey in Nigeria^15^ on children health, about 16% of children reported having fever in the two weeks preceding the survey. The prevalence of fever was highest among children from the poorest households (17%), compared to 15.8% among the middle households and lowest among the wealthiest (13%) (p<0.0001). Of the 3,110 respondents who had bed nets in their households, 506(16.3%) children had fever, while 2,604(83.7%) did not. (p=0.082). In a multilevel model adjusting for demographic variables, fever was associated with rural place of residence (OR=1.27, p<0.0001, 95% CI: (1.16, 1.41), sex of child: female (OR=0.92, p=0.022, 95% CI: 0.859, 0.988) and all age categories (> 6 months), whereas the effect of wealth no longer reached statistical significance.

### Occupation and Migration

Poor households often earn their livelihoods from multiple sources. For example, farmers in Lao People’s Democratic Republic and the Philippines tend to increase their income with non-timber products collected in nearby forests.^16^ Studies have demonstrated a significant link between regular work in the forest and increased risk of malaria also in Africa.^13^,^16^ Among households in the village with bed nets, sleeping in the forest regularly (without a bed net) was associated with an eightfold higher risk of malaria. Notably, the risk of malaria among households in the village that did not use bed nets was similar whether or not an individual slept in the forest or not.^16^ In countries with forest malaria, migrants into forested areas are particularly at risk, because they lack immunity to malaria. Migrants might be drawn to the forests for a variety of reasons, and might or might not be predominantly from poor households.

### Migration and the Spread of P. falciparum

While migrants into forested areas tend to be particularly vulnerable to malaria due to their lack of immunity, they also might transport malaria back into malaria-free zones when they return to their homes or search for work in other areas. During the 1990s, for example, many male workers travelled from communities in Thailand to the gem-mining areas of Borai Province in Cambodia. When they returned to their homes in Thailand, malaria tests revealed that some workers had been infected with resistant strains of P. falciparum.^17^

### Malnutrition and Concurrent Infections

Individuals dwelling in poor households are often malnourished. Malnutrition encompasses not just protein-energy malnutrition, but also deficiencies in micronutrients such as iron, vitamin A, iodine and zinc, in particular. Underweight has been identified as a contributing factor in 60% of all child deaths in developing countries.^17^,^18^ Underweight is believed to increase the susceptibility of children contracting malaria for various reasons, including reduced immunity. Evidence strongly suggests that micronutrient deficiencies and general under nutrition increase the burden of malaria morbidity and mortality.^19^ Individuals in poor households are more likely than those in better-off households to suffer from concurrent infectious and parasitic diseases in addition to malaria.

### Inequalities in Access to Prevention for Malaria

**Figure 3:**
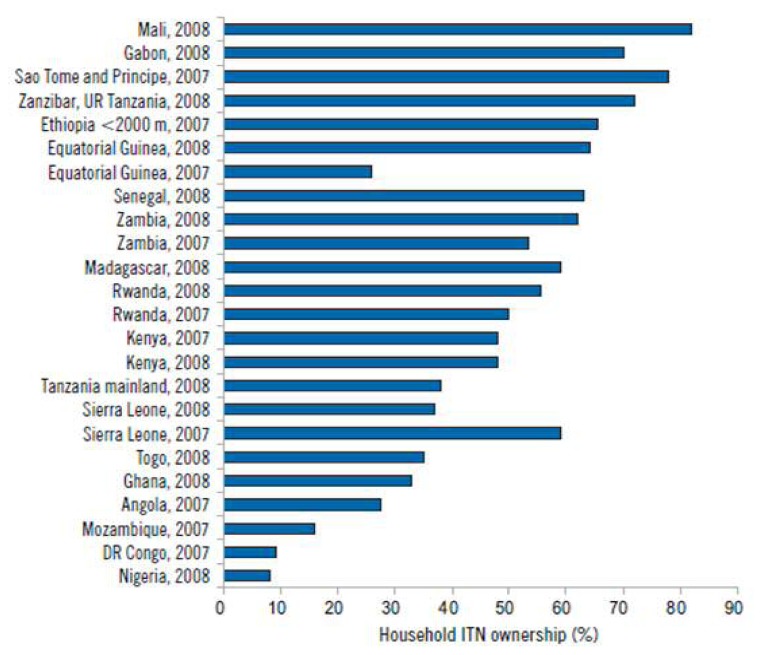
Household insecticide-treated net (ITN) ownership as measured by national surveys, 2007–2008, high-burden WHO African Region countries

Prevention is a key aspect of malaria control, and prompt treatment is considered the most important method of preventing deaths from malaria.^20^ Yet, in the Lao People’s Democratic Republic, for example, only 24% of the population was sleeping under a bed net in 2000, while an estimated 51% of the population of Solomon Islands was sleeping under bed nets in 1999. As discussed below, some evidence suggests that bed net use is higher among non-poor than poor households (Figure 3^5^ shows household insecticide–treated net (ITN) ownership as measured by national surveys. 2007 – 2008 in the high burden WHO African region countries). The same in Tanzania^21^,^22^ where it was shown that poor households living in rural areas spend significantly less on all forms of malaria prevention compared to their richer counterparts, including bed nets, and insecticides. Thus, preventive measures might be missing in poor individuals and households that face greater exposure to malaria than in those that are better off. Inequalities in access to malaria prevention and control might arise from financial and nonfinancial barriers. Separately and together, these barriers can delay or prevent the poor from accessing health care services. For example, based on the findings of a literature review,^12^ poor households are more vulnerable to the effects of malaria than less poor households, possibly because poor households have less access to treatment for malaria than non-poor households. Furthermore, household expenditure on prevention for malaria is more strongly related to income and socioeconomic status than to household expenditure on treatment. However, the cost of seeking treatment for malaria infection is likely to be heavier for poor than for non poor households.^12^

### Economic Barriers

When health services are available, the costs associated with preventive and curative treatment of malaria might deter or prevent the poorer from seeking care. Furthermore, the cost of malaria-related preventive measures has been found to be higher in rural than in urban areas.^23^ Spending on malaria prevention, such as bed nets, appears to be related on household income or socioeconomic status, with better-off households allocating a larger share of their income to malaria prevention than poorer households.^24^ The costs of seeking care can be divided into direct costs (such as fees for services), indirect costs (such as the cost of transportation) and opportunity-loss costs (such as lost wages from time away from work). Although the absolute cost of seeking care as a share of non-food expenditure might be lower for the poor than that for the non-poor, the relative cost of seeking health care is higher.

### Low Education and Knowledge

A general lack of health information and awareness among poor and marginalized groups can greatly reduce the demand for healthcare services. In addition, ethnic minorities might hold beliefs and perceptions about health and illness that influence health seeking. Knowledge of malaria might be lower among poor than non-poor households for several reasons. Information, education and communication (IEC) material for malaria might not reach poor people. Illiterate people and those with low levels of education might be unable to understand written health education materials, such as posters and flyers. Poor households might not have access to radios or television, thereby missing health messages broadcast through these media. Women and ethnic minorities might have even less access to mass media: women tend to be less educated and literate than men, while ethnic minorities can have limited command of the official language of the area or country. Thus, although health information on the cause, transmission and appropriate treatment for malaria might be available in health centers and within villages, such information might not be of any benefit to poor and marginalized groups. Health education delivered through outreach workers likewise might not reach poor households in remote rural villages. In this way, low levels of education can lead to low knowledge of malaria. In turn, such knowledge and perception of malaria is an important factor in determining acceptance and use of malaria prevention and control measures.^2^,^3^

### Socio-Cultural Barriers

Traditional beliefs and practices also can influence whether communities accept and adopt malaria prevention measures and seek treatment. In the northern parts of Viet Nam, for example, ethnic minorities traditionally use bed nets, while those in the Central Highlands traditionally do not. The introduction of bed nets into these areas was more difficult.^3^ In a Mon-Khmer village in Sekong Province of the Lao People’s Democratic Republic, people reportedly used bed nets because of the “nuisance” biting of mosquitoes and other insects, or because they are a status symbol, even though they did not appear to understand the connection between malaria and mosquitoes.^25^

### Inequalities in the Quality of Malaria Treatment

Public sector: many studies from all the poor countries, in Africa and in Asia, have shown that health staffs are reluctant to work in rural and remote health centers.^26^ Further, health posts in remote areas tend to suffer from shortages in essential medicines and equipment 27, which often result in low-quality care and limited confidence in the health care services. Villages near urban centers or along accessible coastal areas enjoy better quality health care than do villages in the remote interior or on isolated stretches of coast.^27^ Patients seeking care during these periods would be given a prescription for anti malarial drugs that could be purchased from a local pharmacy. The irregular supply of free anti malarial drugs, combined with delayed diagnoses, discouraged community members from seeking prompt care for malaria. Furthermore, death from malaria in the Philippines has been attributed to delayed consultation, irregular availability of anti malarial drugs for severe cases in peripheral health centers, and improper treatment from hospital-based physicians.^29^

Private practitioners: Malaria treatment might be offered free in public health centers in many regions. However, patients—including some poor patients—seek care from private practitioners for various reasons, including the perceived poor quality of public health care providers. In areas where antimalarial drugs are available commercially, they can be substandard, counterfeit or outdated.

In a recent study in Kenya,^30^ it has been demonstrated that multiple factors related to affordability, acceptability and availability interact to influence access to prompt and effective treatment. Regarding affordability, about 40% of individuals who self-treated using shop-bought drugs and 42% who visited a formal health facility reported not having enough money to pay for treatment, and having to adopt coping strategies including borrowing money and getting treatment on credit in order to access care. Other factors influencing affordability were seasonality of illness and income sources, transport costs, and unofficial payments. Regarding acceptability, the major interrelated factors identified were provider patient relationship, patient expectations, beliefs on illness causation, perceived effectiveness of treatment, distrust in the quality of care and poor adherence to treatment regimes. Identified availability barriers were related to facility opening hours, organization of health care services, drug and staff shortages. Ensuring that all individuals suffering from malaria have prompt access to effective treatment, remains a challenge for resource constrained health systems. Policy actions to address the multiple barriers of access should be designed around access dimensions, and should include broad interventions to revitalize the public health care system.

## Malaria from a Gender Perspective

A gender approach contributes to both understanding and combating malaria.^31^ Gender norms and values that influence the division of labor, leisure activities, and sleeping arrangements, may lead to different patterns of exposure to mosquitoes for males and females. There are also gender dimensions to accessing treatment and care for malaria, as well as preventative measures such as mosquito nets. A careful understanding of the gender-related dynamics of treatment seeking behavior as well as of decision making, resource allocation and financial authority within households is a key to ensuring effective malaria control programs. Therefore, gender and malaria issues are increasingly being incorporated into malaria control strategies in order to improve their coverage and effectiveness across contexts.^31^,^32^

Women’s household responsibilities such as cooking the evening meal outdoors or waking up before sunrise to prepare the household for the day may put them at greater risk of malaria infection than men in their societies.^32^ Insecticide Treated Net use is also subject to gender norms. Acceptability and use of ITN are strongly linked to culturally accepted sleeping patterns, in which gender plays an important role in who uses the nets. In some instances, young children sleep with their mother and are therefore, protected by her net if she has one. Or, if a household only has one net, priority may be given to the male head of household as he is often considered the primary breadwinner. In other contexts, men have very little access to ITN if they sleep predominantly outside. Hence, understanding how gender patterns of behavior influence exposure to mosquitoes, including use of ITN, can assist in developing more effective recommendations for prevention of malaria infection. For control measures to be effective, health officials have to look at ways of addressing women’s relative lack of power and financial resources. Equally important is the need to target men for malaria control education and sensitization.^32^

Malaria is also a particular problem for pregnant women and adolescent girls^33^. In most endemic areas of the world, pregnant women are the main adult risk group for malaria. They are four times more likely to suffer attacks of symptomatic malaria than other adults. Malaria in pregnancy may quicken severity in patients with drug resistant parasites, anemia, endemic poverty, and malnutrition, like shown recently also in India.^34^ There is, therefore, urgent need to streamline malaria control strategies to make a difference in tackling this grim scenario in human health.

## The Effects of Malaria on Poverty

Malaria might cause and perpetuate poverty at the household level in a number of direct and indirect ways. As outlined above, the total costs of malaria include the direct, indirect and opportunity costs of falling ill and seeking treatment for malaria. Households suffer significant costs when a household member is sick with malaria. The direct and indirect costs of malaria might be substantial, further impoverishing poor households.

Mean direct cost of seeking care for malaria was estimated at 2%–2.9% of household income.^35^ Yet that might mask important economic inequalities In Malawi, for example, annual spending for malaria treatment accounted for 32.1% of average annual income among poor households and only 4.7% of annual income among better-off house-holds.^24^ The cost of malaria to poor households can be especially severe when the sick individual is a productive member of the household, particularly the primary income-earner. Other household labor might be diverted from income-generating activities to care for sick family members. Reduced productivity and time away from work reduce household income. According to studies from Africa, the cost of lost labor from malaria illness might account for more than 75% of the total household cost of malaria.^36^,^42^

However, such strategies require shifting labor away from other productive activities or pulling children out of school. When faced with the cost of seeking treatment for malaria, poor households employ a number of coping strategies. These strategies might include counteracting labor lost due to the illness by employing other household members, including children who are withdrawn from school. Poor households often lack assistance from collateral and social networks in order to raising money for paying for the costs of health care. Therefore, they often end up in borrowing money from local lenders, or might be forced to sell assets, including productive assets such as land and livestock. For example, a study in Cambodia estimated that 40% of new landlessness was due to diseases.^37^ Taking children from school and selling productive assets can deprive households of future income streams. Some evidence suggests that malaria also leads to lower labor productivity by contributing to the prevalence of anemia among poor households. Further, malaria also might contribute to malnutrition and low birth weight. This, in conjunction with reduced learning among children who suffer repeated episodes of malaria, might lead to lower levels of human capital among households in malaria-endemic communities.^38^

When the costs borne by individuals and households suffering from malaria are aggregated at the national level, the loss to development and economic growth is substantial. Estimates from cross-country regressions suggest that malaria morbidity might reduce annual per capita growth by 0.25 percentage points for the most affected countries. Conversely, lower malaria morbidity was also associated with lower poverty ratios and greater access to health care in rural areas 39. A second study estimated that the rate of economic growth in countries with malaria was 1.3% lower per year than in countries without malaria from 1965 to 1990 (controlling for other factors that might influence economic growth). A fivefold difference in Gross Domestic Product (GDP) has been observed between malarial and non-malarial countries, which had an average GDP per capita in 1995 of $1,526 and $8,268, respectively.^40^ Such reductions in GDP might reflect lower rates of savings and investments among households that must spend their income on preventing and treating malaria at the cost of reduced productivity.

### Malaria and Disability

A further condition that can complicate relationship between Malaria and poverty is disability. A very recent study performed in Malawi 41 on disability and poverty has generated information on disabling effects of cerebral malaria as a consequence of poverty. Malawi is among the countries in the world where malaria causes serious health problems. The whole population is at risk. In 2004, about 33% of all children in the district were estimated to have had malaria. Malawi’s population is among the poorest in Africa. Over half the 15 million population is food insecure and dependent on rain-fed smallholder agriculture. Those who live along the lake shore supplement their diet and income by fishing from small boats. While Malawi’s National Statistical Office indicates that 39% are living below the poverty line, Palmer 42 claims that as much as 65% of the population is unable to meet their daily consumption needs.

The informants in this study are known to the health services as malaria survivors. However, they do not receive health services for the disabling after-effects because poverty in most cases has prevented them from seeking such help.

### Cognitive Sequelae of Cerebral Malaria in Children and their Social Impact

A recent study^43^ performed in Uganda, was aimed to describe functional and behaviour deficits in children with severe impairments following cerebral malaria and the patterns of these deficits. The authors found that deficits in motor function, behaviour, vision, speech and hearing or epilepsy were major long-term sequelae. Two main patterns were observed: a) immediate and, b) late-onset deficits. Some deficits (e.g. blindness) resolved, others (e.g. loss of speech) showed little improvement over the follow-up period, while some (e.g. behaviour problems) developed long after exposure. The pathogenesis of these sequalae is unclear but hypoxic neural injury, secondary to parasite sequestration in the cerebral capillary bed during the acute disease, may play a part. This study also describes hearing deficits after cerebral malaria and the poorer prognosis of some sequelae such as loss of speech. Thus, the effects of cerebral malaria are beyond the deaths it is reputed to cause; it may be responsible for impairments in hundreds of thousands of children in the malaria-endemic areas of sub-Saharan Africa. Many of these children may be missing out on school with enormous socio-economic consequences. The stress of managing children with severe impairment and behaviour difficulties may also be putting a lot of strain on family relationships. Strategies for follow-up and continuing care, rehabilitation and family counselling should therefore be developed and incorporated into care protocols.

## Conclusions

Many con-causes help malaria to resist like one the worse killer in the world, and all of them have relationships with poor economic and social conditions. In this point of view the better way to win the battle against malaria is to improve the economic level of populations living in many countries of Africa and Asia together with the synthesis of a good vaccine.

The current global malaria elimination campaign calls also for a health systems strengthening approach to provide an enabling environment for programs in developing countries. In order to realize the benefits of this approach it is vital to provide adequate investment in the ‘people’ component of health systems and understand the multi-level factors that influence their participation. The challenges of strengthening this component of health systems are evident, as is the importance of ensuring that current global malaria elimination efforts do not derail renewed momentum towards the comprehensive primary health care approach.^44^

This is true for other diseases of poverty in order to harmonize efforts at building ‘competent communities’ for communicable disease control and optimizing health system effectiveness.

Unless additional efforts are directed towards addressing access barriers among the poor and vulnerable, malaria will remain a major cause of morbidity and mortality in sub-Saharan Africa and many regions of Asia.

**Figures and Tables below are taken from the following Articles.** Table 1, Figure 2 and Figure 3, are from WHO World Malaria Report 2009, in the bibliography entry n.5, available at ww.WHO.int.

Figure 1 is taken from: Pattanayak S., K. Dickinson, Corey C., Murray B., Sills E., R. Kramer "Deforestation, malaria, and poverty: a call for transdisciplinary research to support the design of cross-sectoral policies" Sustainability: Science, Practice, & Policy 2, 2, 2006 available http://ejournal.nbii.org, in our references n. 6.

## Figures and Tables

**Table 1:** Estimated numbers of malaria cases (in millions) and deaths (in thousands) by WHO Region, 2008

WHO REGION	CASES				DEATHS			
	*Point*	*Lower*	*Upper*	*P. falciparum* (%)	*Point*	*Lower*	*Upper*	Under 5 (%)
AFR	208	155	276	98	767	621	902	88
AMR	1	1	1	32	1	1	2	30
EMR	9	7	11	75	52	32	73	77
EUR	0	0	0	4	0	0	0	3
SEAR	24	20	29	56	40	27	55	34
WPR	2	1	2	79	3	2	5	41
**Total**	**243**	**190**	**311**	**93**	**863**	**708**	**1003**	**85**
